# Evidence of Second Canal between Permanent Mandibular Central and Lateral Incisors in China; a Systematic Review on CBCT Studies

**DOI:** 10.1155/2020/8849609

**Published:** 2020-12-03

**Authors:** Nyan M. Aung, Kyaw K. Myint

**Affiliations:** Department of Oral Biological Science, University of Dental Medicine (Mandalay), 05041 Mandalay, Myanmar

## Abstract

**Introduction:**

Evidence of second canal in permanent mandibular incisors is frequently questioned in dentistry. The difference in evidence between the two teeth is an interesting argument across different countries and ethnicities. So the aim of the systematic review was to investigate the evidence of second canal between permanent mandibular central and lateral incisors in China.

**Materials and Methods:**

The papers were selected from the electronic databases and hand searching according to inclusion and exclusion criteria. All qualified studies were judged by the reviewers. The selected studies were checked with Joanna Briggs Institute Critical Appraisal tool for prevalence studies. Finally, three studies were selected for the review and meta-analyses. The proportion of the second canal with its confidence interval and forest plot for the meta-analyses were calculated.

**Results:**

The evidences of second canal in permanent mandibular central and lateral incisors in China were 5.6% and 14.1%. Only one study reported bilateral symmetry of the second canal as 58.7% and 76.1% in the two types of teeth. Out of all canal anatomies, Vertucci's type ΙΙΙ was dominant comprising 4.1% and 11.2% together with other second-canal types comprising 1.4% and 3% in permanent mandibular central and lateral incisors. When the proportions were meta-analyzed, mandibular central incisors had been less numerous OR = 0.35 [0.31, 0.40], 0.33[0.28, 0.39], and 0.42 [0.22, 0.79] in the evidence of second canal, of Type ΙΙΙ, and of other types except Type ΙΙΙ than mandibular lateral incisors in China. Out of all second-canal anatomies, Type ΙΙΙ presented 72.5% and 78.9% along with other second-canal types comprising 24.3% and 21.1% in the two teeth in China. *Discussion.* There was the evidence of second canal which deviated toward the permanent mandibular lateral incisor also in case of bilateral symmetry, the prevalence of Vertucci's Type ΙΙΙ, and other second-canal types out of all canal anatomies.

## 1. Introduction

Long-standing teeth in the oral cavity are permanent mandibular incisors even when the person is suffering from being partially edentulous. Human dentition is heterodont and diphyodont. So there are two types of permanent mandibular incisors, central and lateral as a general rule.

Although the external morphology of permanent mandibular central and lateral incisors is differentiated mostly through the incisal aspect of the teeth, the internal morphology is interested to be investigated for both preclinical anatomies of the teeth and clinical prognostic indicator. Most prevalence proportion of internal morphology of permanent mandibular incisors is Vertucci's Type Ι, one canal and one foramen configuration. The obvious differences can be found in second modes of Vertucci's classification across the world. In this case, Vertucci's Type ΙΙ and Type V were the second prevalence types of Italian and Turkish population [1, 2]. In contrast, Type ΙΙΙ next to Type Ι was commonly investigated in Chinese populations [3, 4].

However, incidence of second canal can be considered as a coincidence of anatomical and clinical importance because the missed canal was the fourth common of requiring endodontic retreatment [5]. As a result, the most frequent clinical question on the incidence of second canal in permanent mandibular incisors should be emphasized in routine clinical practice.

And the Chinese population in China was counted to nearly 1.40 billion in 2019 [6]. Also, 50 million of oversea Chinese population was recorded in 2012 [7]. In Myanmar, Chinese was estimated to be 3% of Burmese population in 2017 [8]. Yangon, Mandalay, and Taunggyi are the regions with significant Burmese Chinese population [9]. Myanmar is standing as the seventh country where oversea Chinese population was significant.

There are plenty of evidences of Cone Beam Computed Tomography which investigated root canal anatomy of permanent mandibular incisors or permanent mandibular anterior teeth and were conducted in China [3, 4, 9]. These studies pointed out different degree of prevalence of second canal in Chinese population in different regions in China. And there were a few systematic reviews undergone for the internal morphology of the teeth [10–12]. However, no systematic review and meta-analysis on the evidence of second canal between permanent mandibular central and lateral incisors was undertaken.

So the aim of the study was to investigate the evidence of second canal between permanent mandibular central and lateral incisors in China. And the objectives were

1. To identify bilateral symmetry of second canal between permanent mandibular central and lateral incisors in China
2. Out of all canal anatomies, to find the evidence of Vertucci Type ΙΙΙ between permanent mandibular central and lateral incisors in China
3. Out of all canal anatomies, to analyze the evidence of other types of second-canal configurations except Type ΙΙΙ between permanent mandibular central and lateral incisors in China
4. Out of all second-canal anatomies, to evaluate the evidence of Vertucci Type ΙΙΙ between permanent mandibular central and lateral incisors in China
5. Out of all second-canal anatomies, to examine the evidence of other types of second-canal configurations except Type ΙΙΙ between permanent mandibular central and lateral incisors in China.

## 2. Materials and Methods

### 2.1. Inclusion and Exclusion Criteria

The characteristics of the included studies were in vivo prevalence and retrospective studies implemented with CBCT (Cone Beam Computed Tomography), Vertucci's classification used and implemented in China, and being published from 2010 to 2019 (10 years). Diagnostic studies, treatment modality, animal studies, age estimation studies, case reports, the studies on other anatomy, such as incisive canal, and TMJ, systematic reviews, and studies with no calibration on examiners were excluded. Patients, aged 15 years and above, requiring CBCT for dental treatment and having permanent mandibular central and/or lateral incisors were eligible. Importantly the characteristics of the teeth were fully developed root, untreated and having no periapical pathology.

### 2.2. Search Strategies

For clarification of the included studies, we considered different search strategies in PubMed, Science Direct, Willey Online Library, and Goggle from 1^st^ April 2020 to 30^th^ April 2020. MeSH and search terms were “Cone Beam Computed Tomography,” “Tooth,” “Root Canal,” “Anatomy,” “Morphology,” “China,” “Chinese,” “Mandibular,” “Incidence,” “second canal,” “anterior” and “Incisors.” Searching was restricted to abstracts and 10 years' duration by filter. No language restriction was needed. And hand searching was completed on reading textbooks, journals, theses, and posters of the conferences concerning internal canal anatomy of teeth.

First, total 348 studies resulted from search. Three hundred and forty-three studies were excluded by reading the titles and abstracts. Of these, 107 studies were excluded due to treatment modality and intervention studies. Then 75 were excluded due to other anatomies such as external morphology, incisive canal, and TMJ. Also, 53 animal studies and 36 disease prevalence studies were excluded. Continuously, 32 diagnostic studies, 11 anthropological studies, 10 case reports, 4 outside China, 4 in vitro studies, 3 age estimations, and 3 studies of dose-response, deciduous, and mixed dentition were also excluded. Subsequently, 2 studies in only one type of mandibular incisors were investigated and 3 systematic reviews were also excluded. The remaining five studies were downloaded and full-texts read. Finally, 2 studies were excluded because the examiners were not calibrated. Three studies [9, 13, 14] were included for the systematic review and meta-analyses. One of the selected 3 studies [9] was translated from Chinese to English by Goggle Translator.

### 2.3. Working Definition

Vertucci's classification [15] for root canal morphology is as follows: Type I, a single canal from pulp chamber to the apex (1-1 configuration); Type II, two separate canals leaving the chamber but merging short of the apex to form a single canal (2-1 configuration); Type III, a single canal that divides into two and subsequently merges to exit as one (1-2-1 configuration); Type IV, two separate canals from orifice to the foramen (2 configuration); Type V, a single canal leaving from the orifice and separating two canals at the apex (1-2 configuration); Type VII, from a single canal that separate, combines, and pierces as two distinct canals out of the apex (1-2-1-2 configuration); Type ΙΧ, three distinct canals, merging and existing into one canal at the apex (3-1 configuration); and Type Χ, two canals merging to one and then dividing again as two and then merging into one canal to the apex (2-1-2-1 configuration). Types ΙΧ and Χ were additionally classified by Gulabivala et al. [16] who modified Vertucci's classification. In this systematic review, Vertucci's classification was categorized into two groups. Type Ι was categorized as one canal and other classes as two or second-canal. Types ΙΙ, ΙΙΙ, ΙV, V, VΙΙ, ΙΧ, and Χ were altogether set as the evidence of second canal (event). As a result, the categorical variables of Vertucci's classification were dichotomized into Vertucci's Type Ι (one canal) and Vertucci's Types ΙΙ, ΙΙΙ, ΙV, V, VΙΙ, ΙΧ, and Χ (second canal/event) in this systematic review.

### 2.4. Data Analysis

After the studies were selected according to the characteristics of the participant and inclusion and exclusion criteria of the studies, disagreements that occurred were resolved by discussion of the two review authors. The capacity of all publications was justified with the Joanna Briggs Institute (JBI) [17] Critical Appraisal Checklist for prevalence studies. Then the data were extracted and analyzed. Data extraction form included study, types of Vertucci's classification, and bilateral symmetry of second canal.

Weighted mean values with standard deviations (SD) of both inter- and intraexaminer reproducibility across the selected studies were calculated by IBM SPSS. Then the evidences of second canal according to types of Vertucci's classification were extracted. The proportions of the outcome of interest (percentage of second canal) along with its 95% Confidence Intervals were calculated by R package (EZR [32-bits]). Powers for comparison between the two proportions were calculated by R package (EZR [32-bits]) before meta-analyses and subgroup analyses. If there was not enough statistical power, the analyses were not done because at least 80% of power was favorable to decrease *β*-error. And Bonferroni's approach [18] was used to reduce inflated *α*-error when multiple subgroup analyses were made. The equation in which level of significance was divided by number of subgroups was applied. After all, meta-analyzes were performed by Review Manager 5.3 (RevMan 5.3). Mantel-Haenszel OR (Odds Ratio) together with 95% CIs (Confidence Intervals) of second canal between the two teeth was computed and random effects or fixed effects models were used on whether the heterogeneity were present or not. The statistical unit was the tooth and not the patient. Finally, subgroup data analysis was intended to be conducted for both Vertucci's Type ΙΙΙ and other types of second-canal configuration except Vertucci's Type ΙΙΙ (Vertucci's Types ΙΙ, ΙV, V, VΙΙ, ΙΧ, and Χ) out of all second-canal anatomies (objectives 4 and 5).

All three selected studies agreed with the aim and four objectives of the review (objectives 2, 3, 4, and 5). Only one study [9] dealt with the remaining objective of the review (objective 1). The remaining two [13, 14] selectively reported bilateral summary of second-canal. Subsequently, three meta-analyses were conducted in accordance with the aim and objectives 2 and 3. Subgroup analyses were intended to be undertaken for objectives 4 and 5. All three studies were conducted for three meta-analyses (aim and objectives 2 and 3) due to the adequate power (Power = 100%) and *P* = 0.02 was calculated with Bonferroni's alpha allocation [18] and set as significance.

Bilateral symmetry of second canal, and out of all second-canal anatomies (subgroup analysis), the odds of Vertucci's Type ΙΙΙ between permanent mandibular central and lateral incisors, and the evidences of other types (Vertucci's Types ΙΙ, ΙV, V, VΙΙ, ΙΧ, and Χ) except Type ΙΙΙ between permanent mandibular central and lateral incisors (objectives 1, 4, and 5) were not conducted for meta-analysis and subgroup analyses because the statistical powers for the proportions of each finding were 62.7%, 53.7%, and 17.3%.

## 3. Results

Total number of teeth was 11,176. And total number of participants was 2944.

Weighted mean values with standard deviations (SD) of interexaminer and intraexaminer reproducibility (kappa) of all three studies for types of Vertucci's classification were calculated (see Table 1). The mean proportion of JBI score for all the studies was 66.7% [CI-43%–85.4%].

**Figure 1:**
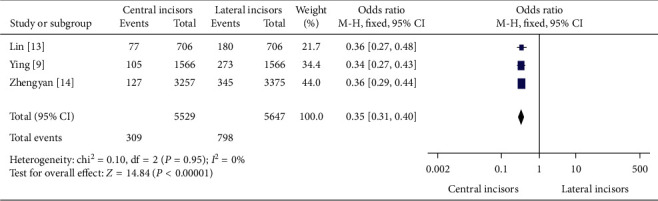
The evidence of second canal between permanent mandibular central and lateral incisors in China.

In Figure 1, the evidence of second canal between permanent mandibular central and lateral incisors in China was meta-analyzed. 309 (5.6% [95% CI-5%–6.2%]) out of 5529 permanent mandibular central incisors had second canal anatomy whereas 798 (14.1% [95% CI-13.2%–15.1%]) out of 5647 permanent mandibular lateral incisors had so.

Only one study [9] reported bilateral symmetry of second canal. From the study, 37 (58.7% [95% CI-45.6%–71%]) out of 63 permanent mandibular central incisors and 108 (76.1% [95% CI-68.2%–82.8%]) out of 142 permanent mandibular lateral incisors had second canal configurations in the balanced distribution. The finding was not meta-analyzed.

**Figure 2:**
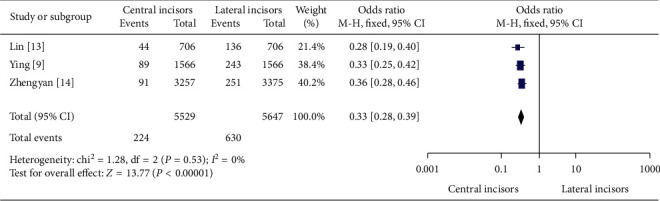
The evidence of Vertucci's Type ΙΙΙ canal configurations between permanent mandibular central and lateral incisors in China.

In Figure 2, out of all canal anatomies, the evidence of Vertucci's Type ΙΙΙ canal configurations between permanent mandibular central and lateral incisors in China was analyzed. All three studies were included in the meta-analysis. 224 (4.1% [95% CI-3.5%–4.6%]) out of 5529 permanent mandibular central incisors and 630 (11.2% [95% CI-10.3%–12%]) out of 5647 permanent mandibular lateral incisors comprised Vertucci's Type ΙΙΙ canal configurations.

**Figure 3:**
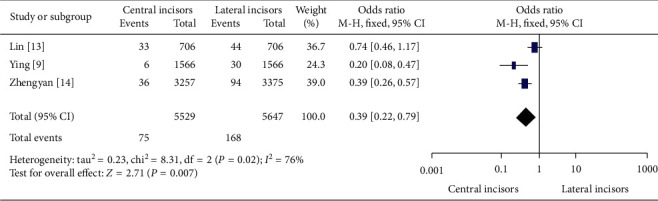
The evidence of second canal configurations (Vertucci's Types ΙΙ, ΙV, V VΙΙ, ΙΧ, and Χ) except Vertucci's Type ΙΙΙ between permanent mandibular central and lateral incisors in China.

In Figure 3, out of all canal anatomies, the evidence of second-canal configuration (Vertucci's Types ΙΙ, ΙV, V, VΙΙ, ΙΧ, and Χ) except Type ΙΙΙ between permanent mandibular central and lateral incisors in China was meta-analyzed. 75 (1.4% [95% CI-1.1%–1.7%]) out of 5529 permanent mandibular central incisors together with 168 (3% [95% CI-2.5%–3.5%]) out of 5647 permanent mandibular lateral incisors had the remaining types of Vertucci's classification except Vertucci's Type ΙΙΙ.

Out of all second-canal anatomies (Vertucci's Types ΙΙ, ΙΙΙ, ΙV, V, VΙΙ, ΙΧ, and Χ), Type ΙΙΙ acquired 224 (72.5% [95% CI-67.2%–77.4%]) out of 309 permanent mandibular central incisors and 630 (78.9% [95% CI-76%–81.7%]) out of 798 permanent mandibular lateral incisors.

Out of all second-canal anatomies mentioned above, other types (Vertucci's Types ΙΙ, ΙV, V, VΙΙ, ΙΧ, and Χ) except Type ΙΙΙ acquired 75 (24.3% [95% CI-19.6%–29.4%]) out of 309 permanent mandibular central incisors and 168 (21.1% [95% CI-18.3%–24%]) out of 798 permanent mandibular lateral incisors.

## 4. Discussion

5.6% of permanent mandibular central incisors comprised second-canal anatomy in contrast to 14.1% of permanent mandibular lateral incisors comprising so in this systematic review. And, only one study of this review pointed out that 58.7% of second-canal anatomies possessed bilateral symmetry in permanent mandibular central incisors whereas two-canal lateral incisors had 76.1% of the balanced distribution. Out of all canal anatomies, Vertucci's Type ΙΙΙ was distributed only 4.1% in permanent mandibular central incisors while 11.2% of this was found in permanent mandibular lateral incisors. However, 1.4% and 3% of other second-canal types (Vertucci's Types ΙΙ, ΙV, V, VΙΙ, ΙΧ, and Χ) were investigated in permanent mandibular central and lateral incisors in China. And, Type ΙΙΙ anatomy was the commonly found second canal type in China. As a result, the evidence of second canal was biased in favor of permanent mandibular lateral incisors.

Out of all second-canal anatomies (Vertucci's Types ΙΙ, ΙΙΙ, ΙV, V, VΙΙ, ΙΧ, and Χ), Type ΙΙΙ acquired 72.5% of 309 permanent mandibular central incisors and 78.9% of 798 permanent mandibular lateral incisors. As a contradiction, other types (Vertucci's Types ΙΙ, ΙV, V, VΙΙ, ΙΧ, and Χ) except Type ΙΙΙ acquired 24.3% of 309 permanent mandibular central incisors and 21.1% of 798 permanent mandibular central incisors out of all second-canal anatomies mentioned above.

In the meta-analyses of Figures 1–3, the evidence of second canal was less frequent odds ratio (OR) = 0.35 (95% Confidence Intervals (CI) 0.31 to 0.40; significance, *P* < 0.00001) in permanent mandibular central incisors than permanent mandibular lateral incisors in China. And out of all canal anatomies, Vertucci's Type ΙΙΙ was less abundantly found (OR = 0.33) (95% CI 0.28 to 0.39; significance, *P* < 0.00001) in permanent mandibular central incisors than lateral. Finally, other second canal configurations (Vertucci's Types ΙΙ, ΙV, V, VΙΙ, ΙΧ, and Χ) except Type ΙΙΙ were less numerous (OR = 0.42) (95% CI 0.22 to 0.79; *P* = 0.007) in permanent mandibular central incisors than laterals in China.

The majority of evidences showed the proportion of second canal configuration was more predominant in permanent mandibular lateral incisors than central incisors in China. Of these, Han et al. [3] stated that the second canal encompassed 15.5% of permanent mandibular central incisors along with 27.36% of permanent mandibular lateral incisors in a Chinese subpopulation. This study outranked the occurrence of second canal anatomy in permanent mandibular incisors when comparing with the data of the review. On the other hand, Martins et al. [19] revealed that 5% and 0.4% of permanent mandibular central and lateral incisors acquired this anatomy in a Chinese population in Suzhou. In addition, Liu et al. [4] investigated that 8.9% and 17.5% of permanent mandibular central and lateral incisors presented the existence of second canal anatomy. This is in agreement with the findings of the review.

Apart from China, Saati et al. [20] stated that 15.5% and 21.8% of second canal were found in permanent mandibular central and lateral incisors in Iranian population. Shemesh et al. [21] revealed that 40.5% and 37.9% of permanent mandibular central and lateral incisors in Israeli population were counted as second canal whereas Valenti-Obino et al. [1] found that 45% and 43% of the second canal comprised in permanent mandibular central and lateral incisors. These findings are contradictory to the data of the study.

There are a few studies which validated bilateral symmetrical distribution of second canal although this anatomical significance is supporting clinical importance. There was no significant difference between permanent mandibular central (69.8%) and lateral incisors (68.7%) in Israeli population [21] in case of bilateral symmetrical distribution of second canal in contrast to the findings of the review.

Several lines of evidences including this systematic review suggested that Vertucci's Type ΙΙΙ second-canal configuration was dominant out of other types of Vertucci's classification in Chinese population [3, 4, 9, 13, 14]. Of all second-canal morphologies, Type ΙΙΙ belonged to more than 40% and nearly 60% of cases by Han et al. [3], over 59% of cases by Liu et al., [4] for both types of teeth, over 57% and 75% of cases by Lin et al. [13], 89% and more than 93% of cases by Ying et al. [9], and 72% and nearly 73% of cases by Zhengyan et al. [14] for permanent mandibular central and lateral incisors.

However, in Italian population, more than 76% and 83% of second canal anatomies for permanent mandibular central and lateral incisors were surprisingly investigated as Vertucci's Type ΙΙ^1^. In Brazilian population, 100% of second-canal anatomies of the teeth were found as Vertucci's Type ΙΙ by Estrela et al. [22]. Turkish population [2] had Type V which comprised 64% and more than 61% of second-canal anatomy of permanent mandibular central and lateral incisors. These findings disagree with and are opposite to the statistics of this systematic review.

Moreover, Han et al. [3] stated that second canal except Vertucci's Type ΙΙΙ presented 9.18% of permanent mandibular central incisors together with 11.67% of permanent mandibular lateral incisors in a Chinese subpopulation. This study revealed the occurrence of second canal anatomy except Vertucci's Type ΙΙΙ was escalated in permanent mandibular incisors when comparing with the data of this review. In addition, Lau [4] investigated 3.6% and 7% of permanent mandibular central and lateral incisors presented the existence of Vertucci's Type-ΙΙΙ-off second canal anatomy. This is more than twice to the findings of the review. On the other hand, Martins et al. [19] found that 0% and 4.2% of permanent mandibular central and lateral incisors acquired this anatomy in a Chinese population in Suzhou. The second canal anatomy except Vertucci's Type ΙΙΙ was scarcity in this study but nearly agreeable to the findings of the review.

Most of the systematic reviews were undergone to obtain external validity and generalizability of the study. Nearly all the findings from the meta-analyses had the narrow confidence intervals which reflect increase in statistical power, the large sample size and representativeness of the target population, thereby potentiating the external validity of the study reducing false-negative error. On the other hand, the large sample size potentiates the negligible effect size to become significant and also favoring false-positive error. The *α*- and *β*-error are the opposite sides of the coin.

Overlapping of confidence intervals of the effect size and null value of *I*-squared statistics in two out of all three meta-analyses pointed out homogeneity among the studies. One of the meta-analyses in this review, the evidence of second canal configurations (Vertucci's Type ΙΙ, ΙV, V, VΙΙ, ΙΧ and Χ) except Vertucci's Type ΙΙΙ between permanent mandibular lateral and central incisors in China had shown 76% heterogeneity in *I*-squared uncovering the statistical heterogeneity with broad confidence interval in case of prevalence of Vertucci's Type ΙΙΙ-off second canal among studies. However 50%–90% of *I*-squared statistics may represent the substantial amount of statistical heterogeneity [23].

Moreover multiplicity and *β*-error may have potentials resulting from multiple subgroup analyses and the reduced statistical power. All the studies used in the meta-analysis were prevalence studies. And there was no priori hypothesis to undergo subgroup analysis. So adjusting significance of Bonferroni's approach and power analyses was acquired to reduce both these false-positive and false-negative errors. Jackson et al. [24] stated that two or three subgroup analyses were set as a rule of thumb and could be favorable for clinical trials.

Although Vertucci's classification was globally accepted in most of the previous studies of internal morphology of permanent dentition, it has not been recognized as an ideal one in this field. Filpo-Perez et al. [25] reported 13% of the investigated teeth were not in harmony with Vertucci's classification. Because all categories of root canal anatomy of human dentition found around the world were not found in this classification, additional classes of Gulabivala et al. [16] and new classification approved by Ahmed and colleagues [26] were approved. As a result, Vertucci's classification has deficiency in content validity. To attain this validity, other categories and calibration of observer are needed to be considered in addition to Vertucci's to avoid misclassification bias and observer bias.

In addition, the prevalence studies were included in the meta-analysis which only pointed out the association between covariates, permanent mandibular incisors, and the evidence of second canal, outcome of interest, excluding other independent variables, age and gender. So, these variables are needed to be demonstrated to identify the confounding in data processing. But the selected studies underreported and variation in reporting methods. And some of the selected studies [13, 14] reported outcomes selectively. All of these were prevalence and retrospective on previous CBCT slices. So there was no random allocation of the participants leading to selection bias.

## 5. Conclusion

As a summary, there was the evidence of second canal which was biased toward the permanent mandibular lateral incisor, also in case of bilateral symmetry, the prevalence of Vertucci's Type ΙΙΙ and other types of Vertucci out of all canal anatomies. So Type ΙΙΙ was the most significant type of second-canal configuration in China. And out of all second-canal anatomies, other types of Vertucci classification except Type ΙΙΙ had a slight burden on permanent mandibular central incisors than lateral incisors in China. The authors concluded that there were more obvious variations in internal morphology of permanent mandibular lateral incisors than central in China.

## Figures and Tables

**Table 1:** Weighted means values for both inter- and intraexaminer reproducibility.

Studies	Types of examiner	Interexaminer reproducibility (Kappa)	Intraexaminer reproducibility (Kappa)	Number of calibrated teeth
Lin et al. [13]	Endodontist versus radiologist	0.91	0.92	200
Ying et al. [9]	Endodontist versus radiologist	0.96	—	424
Zhengyan et al. [14]	Investigator	—	0.82	200
Weighted mean value ± SD		0.93 ± 0.02	0.87 ± 0.05	

SD: Standard Deviation.

## Data Availability

Datasets of this systematic review are available from https://www.google.com/url?sa=t&source=web&rct=j&url=https://data.mendeley.com/datasets/68vx62kspc&ved=2ahUKEwiuteiw767tAhVZT30KHUbBBMAQFjAAegQIBBAC&usg=AOvVaw1WDUe4pbWLJJ8iTdn_XmaH.
